# Supplementary material for: Mismatch Between Perceived and Actual Dietary Nutrition in Hospitalized Cardiovascular Patients and Clinicians: A Cross-Sectional Assessment and Recommendations for Improvement
Source: Nutrients. 2025 Aug 13;17(16):2624. doi: 10.3390/nu17162624 (PMC12389558; doi:10.3390/nu17162624)
Supplement: Supplementary file 1 [file nutrients-17-02624-s001.zip › nutrients-3791831-supplementary.pdf]

Supplementary Material

Table S1. NRS 2002 for hospitalized patients in department of cardiology.

Impaired nutritional status		Severity of disease	
Absent<br>Score 0	Normal nutritional status	Absent<br>Score 0	Normal nutritional requirements
Mild<br>Score 1	Weight loss >5% in 3 months or<br>Food intake below 50-75% of normal requirement in preceding week.	Mild<br>Score 1	Hip fracture<br>Chronic patients, in particular with acute complications: cirrhosis, COPD.<br>*Chronic hemodialysis, diabetes, oncology.*
Moderate<br>Score 2	Weight loss >5% in 2 months or<br>BMI 18.5-20.5+impaired general condition or<br>Food intake 25-50% of normal requirement in preceding week.	Moderate<br>Score 2	Major abdominal surgery<br>Stroke<br>*Severe pneumonia, hematologic malignancy.*
Severe<br>Score 3	Weight loss >5% in 1 month (≥15% in 3 months) or<br>BMI <18.5+impaired general condition or<br>Food intake 0-25% of normal requirement in preceding week in preceding week.	Severe<br>Score 3	Head injury<br>Bone marrow transplantation<br>*Intensive care patients (APACHE>10).*

Footnote: BMI, body mass index; COPD, chronic obstructive pulmonary disease; APACHE, acute physiological and chronic health evaluation system

Table S2. Phenotypic and etiologic criteria for the diagnosis of malnutrition in hospitalized cardiology patients.

**Phenotypic criteria**			**Etiologic criteria**	
Weight Loss (%)	Low BMI (kg/m^2)	Reduced Muscle Mass	Reduced Food Intake or Assimilation	Inflammation
>5% within past 6 months, or >10% beyond 6 months	Asia:<br><18.5 if <70 years, or<br><20 if >70 years	Reduced by validated body composition measuring techniques	≤50% of ER > 1 week, or any reduction for >2 weeks, or any chronic GI condition that adversely impacts food assimilation or absorption	Acute disease/injury or chronic disease-relate
	<20 if <70 years, or <22 if >70 years			

Footnote: BMI, body mass index; ER, energy requirements; GI, gastrointestinal

Table S3. Investigation of dietary knowledge and behavior of hospitalized cardiovascular medicine patients.

Questions	Answers	Percent (%)
Are you confused about how to eat scientifically?	Yes	32.52
	No	67.48
Have you received nutrition education?	Yes	56.31
	No	43.69
How do you get dietary nutrition information?	None	23.46
	Doctor	3.70
	TV	11.73
	Network	31.79
	Friends	11.42
	Books	2.16
	Dietician	3.40
	Newspaper	0.93
	Own ideas	11.42
Are there any contradictions or inconsistencies in the dietary nutrition information you receive?	Yes	30.10
	No	69.90
When you get conflicting information about dietary nutrition, which source of information do you prefer to trust?	None	50.38
	Doctor	16.29
	Dietician	15.91
	Network	1.52
	TV	1.14
	Paper	0.38
	Friends	0.76
	Own ideas	13.64
What is your daily physical activity level?	Bedridden	2.91
	Light activity	97.09
	Moderate activity	0.00
	High intensity activity	0.00
Do you think your eating habits are healthy?	Very healthy	25.73
	Healthy	65.53
	General	5.34
	Unhealthy	3.40
	Very unhealthy	0.00
Which of the following supplements are you taking or planning to take?	None	62.88
	Fish oil	5.68
	Coenzyme Q10	3.93
	Protein powder	3.06
	Nutrition powder/liquid	2.62
	Calcium	8.73
	Vitamin D	0.00
	Multivitamins	8.73
	Dietary fiber powder	1.31
	Probiotics	3.06
What foods do you think should not be eaten?	None	39.69
	Pork	0.31
	Beef	1.54
	Mutton	2.46
	Chicken	0.00
	Duck	0.31

	River fish	1.54
	Marine fish	16.31
	Shrimp	16.92
	Carb	16.92
	Egg	1.23
	Egg yolk	1.54
	Dairy product	0.62
	Soy milk	0.62
Do you think diet helps you manage your disease?	Yes	91.26
	No	8.74
Have you heard of the Dietary Guidelines for Chinese Residents of the Chinese Nutrition Society?	Yes	17.96
	No	82.04
Are you willing to improve your eating habits under the guidance of a dieticians?	Yes	84.94
	No	15.05
How do you feel about your appetite?	Very good	27.32
	Good	61.95
	General	7.32
	Bad	1.95
	Very bad	1.46
What is the main reason that affects your appetite?	None	30.58
	Food taste	8.59
	Disease-related symptoms	14.09
	Psychological stress	5.50
	Monotonous diet	6.53
	Nausea	1.37
	Vomit	2.06
	Anorexia	0.00
	Taste changed	0.00
	Constipation	12.71
	Abdominal distension	2.06
	Diarrhea	1.37
	Pain	2.06
	Dyspepsia	11.34
	Oral disease	1.72
